# Editorial: IgY technology: theory, technical aspects, applications, and innovations

**DOI:** 10.3389/fimmu.2023.1267926

**Published:** 2023-08-18

**Authors:** Xiaoying Zhang, Murtala Bindawa Isah, Mei Dang, Patricia M. Morgan, Marcin Sienczyk, David Bradley, Pablo Chacana

**Affiliations:** ^1^ College of Biological Science and Engineering, Shaanxi University of Technology, Hanzhong, Shaanxi, China; ^2^ Department of Biomedical Sciences, Ontario Veterinary College, University of Guelph, Guelph, ON, Canada; ^3^ Department of Biochemistry, Faculty of Natural and Applied Sciences, Umaru Musa Yar’adua University Katsina, Katsina, Nigeria; ^4^ School of Natural Sciences, University of Galway, Galway, Ireland; ^5^ Division of Organic and Medicinal Chemistry, Faculty of Chemistry, Wrocław University of Science and Technology, Wroclaw, Poland; ^6^ School of Medicine & Health Sciences, University of North Dakota, Grand Forks, ND, United States; ^7^ Instituto de Patobiología, Instituto Nacional de Tecnología Agropecuaria, Nicolas Repetto y Los Reseros S/N, Hurlingham, Buenos Aires, Argentina

**Keywords:** IgY technology, IgY product, publication, commercialization, immunoglobulin Y, egg yolk antibody

Immunoglobulin Y (IgY) from eggs has gained increasing recognition within the scientific community and biotechnology industry. The potential of IgY technology for innovation continues to attract researchers and investors. The first comprehensive monograph on important IgY laboratory protocols, edited by Schade and others, was published in 2001 (1). In the concluding parts of the book, Professor Schade laments on the lack of popularity of hen eggs as antibody sources, which he attributed to the lack of experience and information in using IgY antibodies. Since then, the first comprehensive monograph on IgY technology, edited by Professors Zhang, Vieira-Pires, Morgan, & Schade (2), was published recently and IgY-related publications (**Figures 1A, B**) tripled (3), with each publication further highlighting a potential aspect in which IgY proves to be an excellent antibody source. Similarly, patent registrations and the development of commercial IgY products have increased, collectively expanding the horizons of IgY technology. IgY technology demonstrates superiority over conventional approaches in five frontiers. These frontiers encompass enhanced animal welfare and ethical considerations, abundant IgY content in eggs, the phylogenetic divergence between avian and mammalian immune responses enabling IgY production against conserved mammalian antigens, the molecular organization of IgY genes in birds facilitating rapid gene cloning, and finally, the molecular properties of the IgY molecule itself, including its non-binding affinity to mammalian Fc receptors, mammalian rheumatoid factor, and mammalian complement proteins.

**Figure 1:**
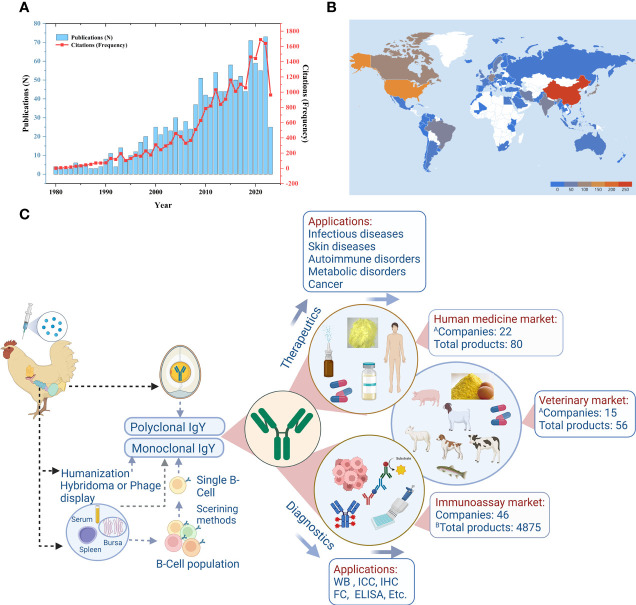
Temporal, geographic status of IgY research and current market landscape of IgY products. **(A)** Number and citation frequency of IgY-related publications; **(B)** Global distribution of IgY-related studies. The data utilized were extracted from the Web of Science (WoS, Clarivate Analytics) database. The retrieval strategy was developed as follows: the Title (TI) was searched for occurrences of “IgY”, “Immunoglobulin Y”, or “egg yolk antibodies”, while the Author Keywords (AK) were set for the same terms. The language type was restricted to English, and the document type was limited to articles, reviews, books, or book chapters. The search encompassed the time span from 1980 to 2023 (as of 30 June 2023). **(C)** Schematic depiction of the current market landscape of IgY products. Reprinted from the literature (Yakhkeshi et al.).

Within this Research Topic, a collective of 38 authors have made significant contributions through the publication of three research articles, one opinion piece, and three review articles. These comprehensive works delve into diverse facets of IgY technology, highlighting its immense potential as a superior resource for biotherapeutics and diagnostics. This discourse extends to an investigation of the present state of IgY commercialization and the associated legal frameworks.

The strength of IgY as a diagnostic tool was further elucidated in one of the original articles by Larsson et al., which reported the development of a particle-enhanced turbidometric assay for detecting human fibrinogen, kappa free light chain, and cystatin C in serum samples. In the article, the advantage of IgY’s non-reactivity to mammalian rheumatoid factor was evident, as the method relies on specific precipitation of the antibody-antigen complex.

The two other original research articles expand our understanding of the potential of IgY antibodies in treating infectious diseases. Brumfield et al. provide insights into the production of IgY targeting a multi-epitope fusion antigen of *Escherichia coli*, showcasing its ability to inhibit the adhesion of enterotoxigenic *E. coli* to mammalian cells *in vitro*. Conversely, Schwartz et al. conducted an *in vivo* murine urinary tract infection model study, where Balb/c mice infected with *Pseudomonas aeruginosa* were prophylactically treated with specific anti-*P. aeruginosa* IgY. These studies further substantiate the potential application of egg-derived IgY in passive immunization against infectious pathogens, offering the promise of reducing antibiotic usage and the development of drug resistance.

Due to the efficient and rapid production of specific IgY in large volumes, it is highly recommended that IgY be considered as a feasible strategy in combating rapidly spreading respiratory pandemics, such as the coronavirus disease 2019, as emphasized by Wallach in this Research Topic. Wallach delineated a scenario in which IgY could be produced in a cost-effective manner and formulated into nasal sprays and lozenges, which can serve as a prompt preventive measure to be administered initially to frontline workers and subsequently in high transmission settings. This approach would be especially invaluable prior to the availability of vaccines, which, aside from the expedited development of COVID-19 vaccines, typically require several years to produce.

Moreover, El-Kafrawy et al. and Grzywa et al. provide comprehensive reviews on the potential of IgY as an anti-infectious agent. The inherent advantage of IgY lies in its ability to target various antigens of infectious agents, thus minimizing the likelihood of resistance development. The reviews captured the limitations of using IgY as a drug, including challenges in oral delivery due to susceptibility to proteolytic cleavage by pepsin, lack of standardized regulations for clinical-grade IgY production, and the necessity to develop standardized industrial protocols for mass production of clinical-grade IgY. These limitations are well recognized within the IgY research community, explaining the active research field of IgY dosage form formulation.

In response to the demand for industrial-scale IgY production, several companies have emerged over the past few decades to meet this need. Yakhkeshi et al. address this subject in their review, discussing the state of commercial IgY production, covering safety considerations, large-scale production, IgY delivery systems and dosage form design, stability of IgY products, and regulatory requirements for commercializing IgY products. The review identified a total of 27 companies engaged in the production of IgY biotherapeutics, along with an additional 46 companies involved in manufacturing various IgY-based diagnostic products. Moreover, the review highlighted the existence of 80 commercial products at different stages of development for utilization in human medicine, with some of these products already available in the market (**Figure 1C**). This progress that was unimaginable two decades ago signifies a remarkable advancement. Furthermore, the review identified an additional 56 products intended for application in the veterinary field. It is anticipated that there will be a continued upsurge in the production of bioactive, highly pure, and safe IgY drugs, as well as other health-related products that have received approval from relevant regulatory agencies. These advancements will undoubtedly play a vital role in the global battle against diseases.

In conclusion, the collection of articles and reviews presented in this Research Topic shed more light on the immense potential of IgY technology in various aspects of biomedical research and clinical applications. From its diagnostic strength to its therapeutic effectiveness against infectious diseases, IgY has demonstrated remarkable advantages as outlined above. However, challenges such as oral delivery, standardized production protocols, and regulatory considerations remain to be addressed. The ongoing efforts of researchers, coupled with the emergence of commercial IgY production companies, indicate a promising future for the development of bioactive and safe IgY drugs. With its ability to rapidly produce targeted antibodies in large quantities, IgY holds particular promise in the face of rapidly spreading respiratory pandemics. As we continue to explore and refine the potential of IgY, we move closer to harnessing its full power in the global fight against diseases.

## Author contributions

XZ: Writing – original draft, Writing – review & editing. MI: Writing – original draft, Writing – review & editing. MD: Writing – original draft, Writing – review & editing. PM: Writing – review & editing. MS: Writing – review & editing. DB: Writing – review & editing. PC: Writing – review & editing.
